# Palladium-rich plasmonic nanorattles with enhanced LSPRs *via* successive galvanic replacement mediated by co-reduction†

**DOI:** 10.1039/d1ra06109g

**Published:** 2021-12-16

**Authors:** Mariia Ivanchenko, Andrew J. Evangelista, Hao Jing

**Affiliations:** Department of Chemistry and Biochemistry, George Mason University Fairfax Virginia 22030 USA hjing2@gmu.edu

## Abstract

Catalytic transformations under light irradiation have been extensively demonstrated by the excitation of the localized surface plasmon resonances (LSPRs) in noble metal-based nanoparticles. To fully harness the potential of noble metal-based nanocatalysts, it is fundamentally imperative to explore hybrid nano-systems with the most desirable enhanced LSPRs and intrinsic catalytic activities. Pd-containing hollow multimetallic nanostructures transformed from the sacrificial template of Ag *via* galvanic replacement reaction (GRR) offer such ideal platforms to gain quantitative insights into nanoparticle-catalyzed reactions. In this work, we successfully fabricated Pd-rich plasmonic nanorattles by means of co-reduction mediated GRR using CTAC-stabilized Au@Ag nanocuboids as templates and H_2_PdCl_4_ as a Pd precursor in the presence of ascorbic acid (AA) acting as a mild reducing agent. Successive titration of Au@Ag nanocuboids with the Pd precursor in the presence of AA modulates the rate of the galvanic replacement reaction as well as effective diffusion of Pd into the Ag matrix, resulting in increased dimensions and enlarged cavity sizes. Reduction of oxidized Ag^+^ back to Ag^0^ by AA, along with the deposition of Pd to form homogeneously mixed bimetallic layers not only prevents LSPRs peak from damping with increasing Pd content but also ensures the enhanced catalytic activities. Through precise control of added H_2_PdCl_4_ titrant, an unconventional steep increase in extinction intensity accompanied by tunable plasmon resonances shifted towards the NIR spectral region was experimentally observed due to the increasing physical cross-sections and plasmon hybridization in hollow nanorattles. Four colloids of Pd-rich nanorattles obtained by addition of different amounts of the H_2_PdCl_4_ titrant were used as catalysts for reduction of 4-nitrothiophenol in the presence of NaBH_4_ monitored by SERS.

## Abstract 2

Palladium-rich plasmonic nanorattles with enhanced surface plasmon resonances synthesized through successive galvanic replacement mediated by co-reduction.

## Graphical abstract

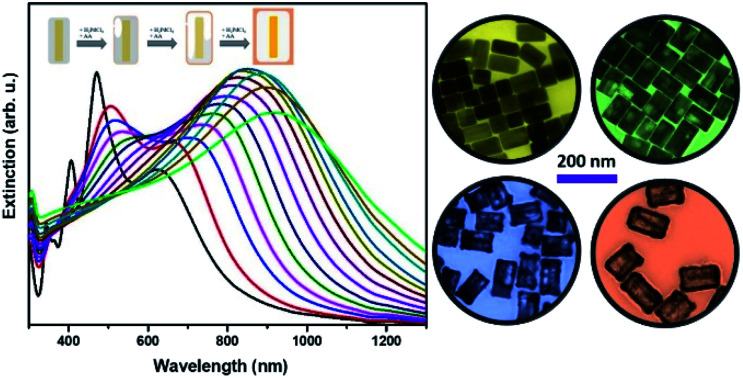

## Introduction

Plasmonic nanocatalysts generally based on noble metal nanoparticles, such as gold (Au),^1–3^ silver (Ag),^4,5^ and copper (Cu),^6^ have now been widely studied in chemical transformations owing to the geometrically tunable optical properties dominated by the collective oscillations of free electrons referred to as localized surface plasmon resonances (LSPRs).^7,8^ However, these nanocatalysts exhibit rather limited catalytic activities, which hinders their practical applications in the real world.^9,10^ Conversely, other noble metal nanocrystals, such as palladium with a myriad of well-controlled morphologies^11–15^ and superior catalytic performances^16–19^ do not support LSPRs excitation in the visible or near-infrared spectral ranges due to unwanted plasmon damping caused by the large imaginary part of the permittivity.^20^ In this context, multicomponent hybrid hetero-nanostructures integrating desired plasmonic and catalytic constituents, where tunable plasmon resonances and the intrinsic catalytic active sites couple synergistically, overcome the limitations and provide ideal platforms to gain quantitative insights into nanoparticle-catalyzed reactions.^21–26^ Besides the conventional wet-chemistry approaches for the fabrication of the multicomponent hybrid nanostructures, such as core–shell nanoparticles,^27–32^ nanoscale galvanic replacement reaction (GRR) has emerged as an alternative yet extremely versatile method to produce multi-metallic hollow particles with tailored interior architectures and enhanced optical and catalytic properties *via* a heterogeneous electrochemical redox process thermodynamically driven by the differences in the reduction potentials of a pair of metals.^33^ Nevertheless, the most majority of the work has focused on the transformation of solid sacrificial template of Ag nanoparticles into Au–Ag hollow nanostructures through the process of galvanic replacement reaction, including nanoboxes, nanocages and nanoframes.^34–42^ Despite the rapid advancement in mechanistic studies of hollow Ag–Pd nanostructures with increased architectural complexity,^43–49^ very few are the examples found in the literature reporting such Pd-containing hollow particles with robust extinction peaks across the broad spectral ranges,^50^ primarily due to the strong plasmon damping when incorporating Pd into the nanoparticles.^51^ Such robustness in the optical behaviors, especially with enhanced plasmon resonances, has only been experimentally observed in Au–Ag hollow nanostructures so far.^52,53^ To fully harness the potential of noble metal-based nanocatalysts, it is fundamentally imperative to explore other hybrid hollow nano-systems with most desirable enhanced LSPRs and intrinsic catalytic activities besides traditional Au–Ag hollow particles, such as Pd-containing hollow nanostructures transformed from the sacrificial template of Ag. Taking into account of the catalytically active characteristics of Pd and the fact Ag nanocrystals exhibiting the strongest plasmon resonances with least damping, greatest optical tunability, and most intense near-field enhancements among all other noble metal counterparts,^54^ the resultant Ag–Pd hollow nanostructures will open new opportunities in various plasmon-enabled applications, such as photocatalysis,^55^ surface-enhanced Raman scattering,^56^ optics,^57^ sensing,^58^ imaging,^59^ photothermal therapies,^60^ and drug delivery.^61^ In our work, we successfully obtained Pd-rich plasmonic nanorattles with enhanced LSPRs *via* successive galvanic replacement reactions mediated by co-reduction in the presence of a mild reducing agent, ascorbic acid (AA). Utilizing anisotropic Au@Ag core–shell nanocuboids as the sacrificial template, certain amounts of palladium precursor (H_2_PdCl_4_) were successively introduced into the reaction solution at ambient temperature to precisely control the addition rates after each round of GRR was completed. Ascribed to the effective diffusion of Pd into Ag matrix and co-reduction of Pd and Ag facilitated by AA, homogeneously mixed Ag–Pd bimetallic walls with optimized distributions in the hollow structures were favourably formed in the early alloying stage of GRR. In conjunction with the increased dimensions and enlarged cavity sizes inside nanorattles during successive GRR evidenced by electron microscopes, an unconventional steep increase in extinction intensity accompanied by tunable plasmon resonances was observed attributed to the increasing physical cross-sections and plasmon hybridization in hollow particles.^62^ To the best of our knowledge, this is the first time well-controlled Pd-containing rattle nanostructures with enhanced extinction through successive co-reduction modified GRR were experimentally obtained. The desirable enhanced LSPRs allowed us to use time-resolved surface-enhanced Raman spectroscopy (SERS) to investigate the excellent catalytic behaviors of such Pd-rich plasmonic nanorattles by probing the kinetics of the reduction of pre-adsorbed 4-nitrothiophenol (4-NTP) by NaBH_4_ under excitation of 785 nm near-infrared (NIR) laser. The detailed understanding will enrich the versatility of current synthetic approaches for rational design of multimetallic hollow nanostructures with unprecedented tunability in compositions, optical, and catalytic properties.

## Results and discussion

Single-crystalline Au nanorods (Au NRs) were used as the core materials, on which Ag shells were epitaxially grown to construct Au@Ag core–shell nanocuboids in presence of ascorbic acid (AA) as a mild reducing agent and cetyltrimethylammonium chloride (CTAC) as surface-capping ligands (Fig. S1B and C†). Au NRs exhibited two extinction peaks located at 505 and 1005 nm, corresponding to the transverse and longitudinal LSPR modes, respectively. The longitudinal LSPR peak of Au NRs underwent hypsochromic shift with increased intensity after Ag deposition because the characteristics of the plasmon resonance modes were dominated by those of the Ag shell (Fig. S1A†).^54^ Au@Ag nanocuboids displayed four distinct peaks in the experimentally measured extinction spectra, corresponding to the longitudinal dipole, transverse dipolar, and two transverse octupolar plasmon resonances, respectively (Fig. S1A†).

**Fig. 1:**
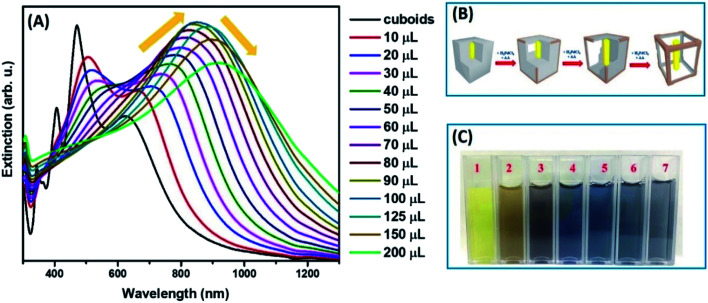
(A) UV-vis-NIR extinction spectra of aqueous colloids of Au@Ag nanocuboids and of Au@Ag/Pd nanorattles obtained using denoted volume of 1 mM H_2_PdCl_4_. (B) Schematic illustration of the stepwise morphological changes in Au@Ag/Pd nanorattles during titration with 1 mM H_2_PdCl_4_ in the presence of AA. (C) Ambient light photograph of colloidal solutions of (1) Au@Ag nanocuboids and (2–7) Au@Ag/Pd nanorattles obtained using (2) 10 μL, (3) 30 μL, (4) 50 μL, (5) 70 μL, (6) 90 μL, and (7) 150 μL of 1 mM H_2_PdCl_4_.

**Fig. 2:**
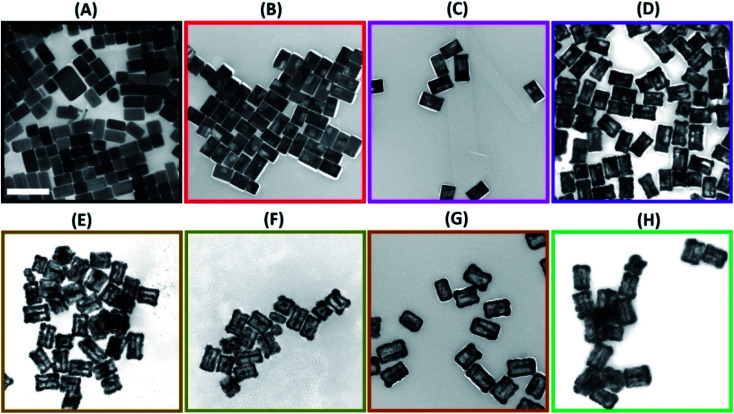
TEM images of (A) Au@Ag nanocuboids and (B–H) Au@Ag/Pd nanorattles obtained using (B) 10 μL, (C) 30 μL, (D) 50 μL, (E) 70 μL, (F) 90 μL, (G) 150 μL, and (H) 200 μL of 1 mM H_2_PdCl_4_. Scale bar corresponds to 200 nm.

To produce Au@Ag nanocuboids with hollowed morphologies, we performed the successive galvanic replacement reactions with H_2_PdCl_4_ in the presence of AA at the ambient temperature. The plasmonic features of colloidal hollow nanostructures obtained upon successive galvanic replacement of Au@Ag core–shell nanocuboids with different amounts of H_2_PdCl_4_ in the presence of AA were monitored by optical extinction spectroscopic measurements (Fig. 1A). As the amount of added H_2_PdCl_4_ increased, the longitudinal dipole plasmon resonance peak progressively red-shifted from 625 nm to 940 nm and significantly broadened. Spectral tunability of the extinction band is governed by the plasmon “hybridization” supported by hollowed nanostructure and geometry. The formation of a homogeneously mixed Ag–Pd bimetallic shell due to effective diffusion and co-reduction of Ag^+^ together with Pd^2+^ by AA inherited the strong LSPRs of original nanocuboids without suppression caused by Pd component in rattle-like nanostructures. More interestingly, the intensity of longitudinal dipole plasmon resonance peak unconventionally raised reaching the maximum for nanorattles titrated with 100 μL of 1 mM H_2_PdCl_4_ during the alloying process occurring in the early stage of GRR (Fig. 1A). The initial steep intensification of extinction peak was attributed to the increase of scattering cross-sections, which was evidenced in transmission electron microscopy (TEM) images (Fig. 2) and size histograms of the nanoparticles (Fig. S2 and S3†). In the later dealloying stage, a decrease in peak intensity of longitudinal dipole LSPRs was observed because of the loss of Ag content in nanorattles and prevailed plasmon damping by the deposited Pd. The transverse dipolar and octupolar plasmon peaks were more significantly damped than the longitudinal LSPRs and they became less distinguishable as their intensities gradually decreased. The gradual morphological evolution of Au@Ag nanocuboids into Au@Ag/Pd nanorattles upon titration with 1 mM H_2_PdCl_4_ is schematically illustrated in Fig. 1B. Such morphological transformation is accompanied by the vivid colour change of colloids from bright yellow to brown, dark blue, and grey as shown in Fig. 1C.

**Fig. 3:**
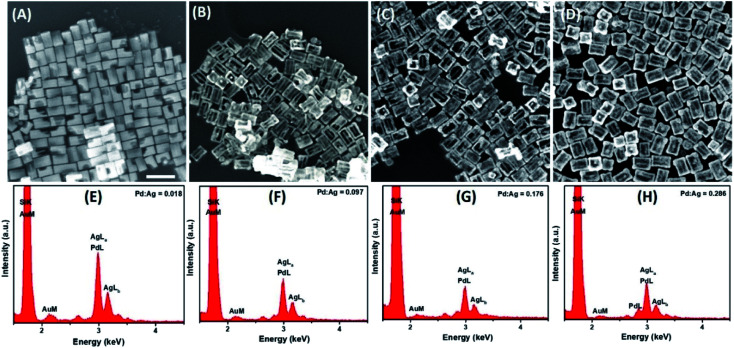
(A–D) SEM images of Au@Ag/Pd nanorattles obtained using (A) 10 μL, (B) 50 μL, (C) 90 μL, and (D) 200 μL of 1 mM H_2_PdCl_4_. Scale bar corresponds to 200 nm. (E–H) Energy-dispersive X-ray spectra of Au@Ag/Pd nanorattles obtained using (E) 10 μL, (F) 50 μL, (G) 90 μL, and (H) 200 μL of 1 mM H_2_PdCl_4_.

**Fig. 4:**
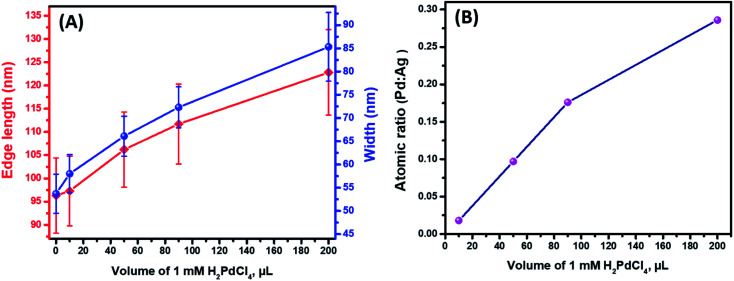
(A) Change in size, edge length and width, of Au@Ag/Pd nanorattles during titration with 1 mM H_2_PdCl_4_. The error bars demonstrate the distribution of Au@Ag/Pd edge lengths and widths. (B) Change in atomic composition of hollowed Ag–Pd shells of Au@Ag/Pd nanorattles with increase of volume of 1 mM H_2_PdCl_4_ solution used.

Further detailed structural and morphological characterization of nanostructures obtained at different stages of GRR was conducted by means of TEM and SEM. Micrographs displayed the abovementioned transformation pattern of Au@Ag nanocuboids into nanorattles with Au NRs inside. Hollowing of Ag shell preferentially initiated from the corners and edges as they provide reactive sites with the highest surface energy^63^ (Fig. 2B and 3A). At the same time, PdCl_4_^2−^ ions were reduced by electrons migrated to the surface of nanoparticles and nonepitaxially deposited on the outer surfaces of the nanocuboids owing to the lattice mismatch between Ag and Pd. As GRR progressed, more thermodynamically stable alloys, instead of segregated Ag and Pd, were formed due to the interdiffusion between two metals sharing the same face-centred cubic (fcc) structure. According to the Joint Committee on Powder Diffraction Standards (JCPDS), bulk gold, silver, and palladium have slightly different lattice parameters, 4.065 Å, 4.079 Å, and 3.859 Å, respectively. X-ray diffraction patterns of Au@Ag nanocuboids and Au@Ag/Pd nanorattles contain four diffraction peaks corresponding to the (111), (200), (220), and (311) planes of the samples (Fig. S4†). However, Au@Ag/Pd nanorattles pattern peaks are slightly displaced to higher 2*θ*, compared to those in Au@Ag nanocuboids pattern. The location of the peak shifted closer toward that of the pure Pd crystal because of the presence of Pd, further indicating the formation of Ag/Pd alloys. Concomitantly, the size of the cavities inside the nanorattles became larger as additional H_2_PdCl_4_ was added (Fig. 2C–H), resulting in the observed redshift of longitudinal dipole LSPRs in the extinction spectra. As determined from TEM pictures, the overall dimension of nanostructures gradually enlarged during morphological evolution (Fig. 2A–H) because of co-deposition of continuously supplied Pd and Ag in the course of successive GRR. More quantitatively, the average lengths of hollow nanostructures increased from 96.3 ± 8.1 nm to 122.8 ± 9.2 nm and average widths changed from 53.7 ± 4.2 nm to 85.3 ± 7.4 nm during successive GRR (Fig. 4A). Additional details about the dimensions and size distribution of hollow nanoparticles at different stages of GRR can be found in the ESI (Fig. S2 and S3†). The increased scattering cross-sections stemming from the enlarged overall dimensions were responsible for the initial steep rise in the intensities of longitudinal dipole LSPRs in the extinction spectra (Fig. 1A). Overall, the co-reduction by AA facilitated the alloying process to generate plasmonic nanorattles with optimized Ag and Pd distribution and enhanced LSPRs. As more H_2_PdCl_4_ was added into the solution mixture, the outer alloyed walls of nanorattles became thicker and much less dense with porous surfaces, as clearly visualized in SEM images (Fig. 3B–D). This porous nature can be most likely interpreted as the consequence of dealloying process occurring at the later stage of GRR, in which Ag atoms are selectively extracted from the alloyed shells and many lattice vacancies left coalesce to generate holes to compensate the increased surface free energies.^64^ It is worth to note that, under these circumstances, the effect of plasmon damping by Pd component prevailed as a result of the loss of Ag content from the alloyed shells, inevitably causing the intensity decrease in longitudinal dipole LSPRs of Pd-rich nanorattles after maximal extinction was reached. Surprisingly, these Pd-rich nanorattles with porous walls were quite stable with no collapse of hollow structures upon the introduction of excessive H_2_PdCl_4_, which is in striking contrast to the fragmentation commonly observed in conventional Ag–Au nanorattles.^52^ Meanwhile, Pd : Ag atomic ratios in the hollow nanostructures were confirmed by energy dispersive X-ray (EDX) spectroscopy analysis to correlate the structures with the chemical compositions (Fig. 3E–H). As previously mentioned, released Ag^+^ ions were co-reduced together with Pd^2+^ ions by AA to form metallic Ag and Pd atoms co-deposited on the surface generating homogeneous Ag–Pd alloy. As the total volumes of H_2_PdCl_4_ added during the course of GRR increased, the proportions of Pd was larger and the Pd : Ag atomic ratios accordingly raised to 0.286 from 0.018 corresponding to 200 μL and 10 μL of 1 mM H_2_PdCl_4_ used, respectively (Fig. 4B).

**Fig. 5:**
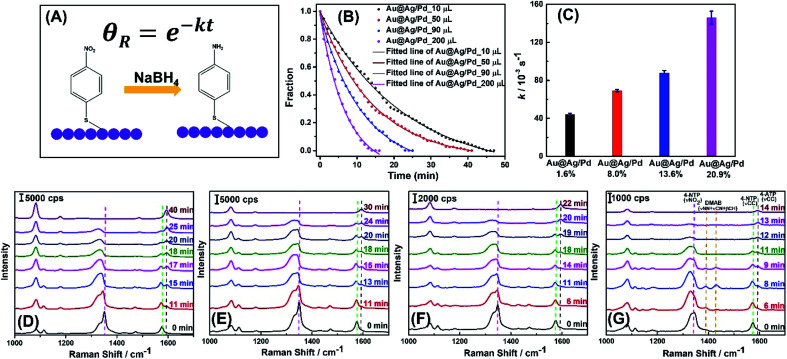
(A) Schematic illustration of the chemical reduction of surface-adsorbed 4-NTP to 4-ATP catalyzed by the Au@Ag/Pd nanorattles that follows first-order rate law. *θ*_R_ denotes the fraction of the reactant, *t* is the reaction time, and *k* is the rate constant to be determined. (B) Fraction of reactant measured at 1348 cm^−1^ in SERS spectra as a function of reaction time during the 4-NTP transformation catalyzed by Au@Ag/Pd nanorattles obtained by titration with 10 μL, 50 μL, 90 μL, and 200 μL of 1 mM H_2_PdCl_4_. (C) The rate constants values of 4-NTP transformation in the presence of Au@Ag/Pd nanorattles obtained by titration with 10 μL, 50 μL, 90 μL, and 200 μL of 1 mM H_2_PdCl_4_. (D–G) Time-resolved SERS spectra collected during the reduction of 4-NTP by NaBH_4_ on Au@Ag/Pd nanorattles obtained using (D) 10 μL (E) 50 μL (F) 90 μL (G) 200 μL of 1 mM H_2_PdCl_4_ and excited at 785 nm.

Such Pd-rich nanorattles with tunable and enhanced LSPRs are promising bifunctional nanomaterials by integrating both SERS and catalytic properties. We used *in situ* SERS to monitor the nanorattle-catalyzed reduction of 4-nitrothiophenol (4-NTP) to 4-aminothiophenol (4-ATP) by NaBH_4_ in the colloidal suspension and then compared the catalytic activities of four nanorattles with increasing Pd : Ag molar ratios, obtained by titration with 10 μL, 50 μL, 90 μL, and 200 μL of 1 mM H_2_PdCl_4_. Through our analysis, we demonstrated that the catalytic activities of nanorattles could be manipulated by varying the amounts of Pd deposited. The strong LSPRs arising from the deposition of Ag and enlargement of cross-sections during alloying process ensured the high detection sensitivity of SERS such that the characteristic Raman peaks of 4-NTP molecules pre-adsorbed on the surface of nanorattles could be clearly resolved under excitation of 785 nm laser. On the other hand, the catalytically active Pd component enriched in nanorattles allowed us to study the kinetics of the reduction reaction. Illustration of transformation to 4-aminothiophenol is shown in Fig. 5A. Typically, four colloidal Au@Ag/Pd nanorattles were incubated with the solution of 4-NTP overnight and SERS spectra at different reaction times were collected right after adding NaBH_4_ into the suspension to initiate the catalytic reaction (Fig. 5D–G). Because NaBH_4_ was added in great excess and the change in its concentration was assumed to be negligible throughout the whole process, the surface reaction followed pseudo-first order kinetics. The integrated rate law is noted on Fig. 5A with *θ*_R_ being the fraction of the reactant, *t* being the reaction time, and *k* is the rate constant to be found. The major characteristic bands in SERS spectrum of 4-NTP are at 1078, 1348, and 1572 cm^−1^ that correspond to C–S, N–O, and C–C stretching vibrations, respectively.^65^ The Raman spectra reflected that as the reaction progressed, the intensities of N–O, and C–C stretching bands gradually decreased, while a new band attributed to the phenol-ring C–C stretching mode of 4-ATP at 1595 cm^−1^ emerged. Besides tracking the reaction progress, the Raman mode *ν*(NO_2_) at 1348 cm^−1^ was used to quantify the fraction of reactant. Based on the collected SERS data, kinetic curves for the four different samples of Au@Ag/Pd nanorattles were plotted (Fig. 5B) and the four rate constants were subsequently calculated to be 0.044 ± 0.0011, 0.069 ± 0.0012, 0.088 ± 0.0025, and 0.146 ± 0.0069 s^−1^ by performing least-square curve fitting to the reaction trajectories (Fig. 5C). It can be concluded that higher Pd : Ag ratios gave rise to higher catalytic activities with larger *k* values. With the acceleration of reaction rates, the time needed for reduction to go completion was significantly reduced from ∼40 min for nanorattles titrated with 10 μL H_2_PdCl_4_ to ∼14 min for those with 200 μL H_2_PdCl_4_ (Fig. 5D and G). Pd concentrations in the sample of nanorattles obtained using 200 μL H_2_PdCl_4_ were analysed and determined to be 21.64 and 19.51 mg L^−1^ by ICP-OES before and after the reaction, further confirming the stability in the structure (Fig. S5 and Table S1†).

Few reports dedicated to trimetallic nanocomposites, containing Au, Ag, and Pd, of various geometries and design were published formerly. Among them, AuAg : Pd concave nanolayers and Au@AgPd core/shell nanoflowers were used in conversion of 4-NTP to 4-ATP monitored by SERS. In comparison to those morphologies, Au@Ag/Pd nanorattles produced increased reaction rates and, therefore, demonstrate higher catalytic activity (Table S2†). Moreover, the values of rate constant for Au@Ag/Pd nanorattles catalyzed 4-NTP to 4-ATP transformation corelate with Pd content in nanocomposite used as catalyst, which is in agreement with previously reported results for AuAg : Pd concave nanolayers. This general trend of better catalytic activities of the nanorattles with a higher Pd : Ag ratio are related to the increased fraction of Pd atoms on the outer and inner surface of the Ag/Pd frame surrounding the Au NR, which serve as catalytic sites, as well as enlarged specific surface areas of those Au@Ag/Pd nanostructures, due to emptying silver content from the inside. Hollow nanoparticles are generated as small vacancies, formed after Ag^0^ was oxidized and diffused into the solution, merge into a single larger void to reduce the surface energy of small pores. The morphological characteristics of nanorattles, such as increased dimensions and enlarged sizes of inner cavity, are determined by the amount of Pd precursor added to Au@Ag sacrificial template during their synthesis. Introducing higher concentration of Pd into the reaction mixture leads to further expansion of inner void, roughening and thickening of outer cage. Presence of interior kinks, edges, high energy surfaces, and increased number of low-coordinated Pd and Ag atoms on the inner surface contribute to higher catalytic efficiency of nanorattles. Also, formation of Ag/Pd frame and hollow structure facilitated better mass transfer comparing to solid architectures, making more active inner sites accessible for the substrate molecules and improving catalytic performance. The results of this study can be used to tune and optimize performance of Au@Ag/Pd nanorattles required by desired application employing a facile and robust synthetic approach.

## Experimental section

### Materials

Hexadecyltrimethylammonium bromide (CTAB, >98.0%), hexadecyltrimethylammonium chloride (CTAC, >95.0%), sodium oleate (NaOL, >97.0%), hydrogen tetrachloroaurate(iii) trihydrate (HAuCl_4_·3H_2_O, 99.99%), l-ascorbic acid (AA, ≥99.0%), silver nitrate (AgNO_3_, >99.0%), sodium borohydride (NaBH_4_, 99.99%), hydrochloric acid (HCl, 37 wt% in water, 12.1 M), palladium(ii) chloride (PdCl_2_, ≥98.0%), 4-nitrothiophenol (4-NTP, technical grade, 80%). All the chemicals were used as received without further purification. The deionized water used in the experiment was ultra-pure (MilliQ, 18 MΩ).

### Synthetic procedures

#### Synthesis of gold NRs

0.6 mL of freshly prepared 0.01 M NaBH_4_ solution was rapidly injected in the mixture of 5 mL of 0.5 mM HAuCl_4_ and 5 mL of 0.2 M CTAB aqueous solution under vigorous stirring. Immediate colour change from yellow to light brown was observed. After 2 minutes stirring at 1200 rpm, seed solution was left undisturbed for 30 minutes. 0.7 g of CTAB and 0.1234 g NaOL were dissolved in 25 mL of water at ∼60 °C. After dissolution, the mixture cooled down to 30 °C and this temperature was maintained during entire synthesis. Then, 1.8 mL of 4 mM AgNO_3_ solution was added, and the mixture was left undisturbed for 15 minutes. When the time was over, 25 mL of 1 mM HAuCl_4_ solution was added, while stirring at 700 rpm. The mixture was stirred for 90 minutes, and the disappearance of yellow colour was observed. The pH value was adjusted by addition of 0.4 mL of concentrated HCl and stirring at 400 rpm for 15 minutes. Next, 0.125 mL of 0.064 M ascorbic acid was injected, while the mixture was vigorously stirred at 1200 rpm. After 30 s, a 0.08 mL of seed solution was introduced into the growth mixture, while maintaining vigorous stirring. The mixture was stirred for 30 s and left undisturbed at 30 °C overnight. Obtained Au NRs were washed by centrifugation at 7000 rpm for 30 min. After the removal of the supernatant, product was redispersed in 10 mL of 0.1 M CTAC.

#### Synthesis of Au@Ag nanocuboids

0.1 mL as prepared Au NRs was diluted to 2 mL with water. While stirred at 65 °C, 0.15 mL of 10 mM AgNO_3_ were added. After stirring at 700 rpm for 10 min, 0.15 mL of 50 mM ascorbic acid were injected. The temperature was maintained during entire synthesis. The reaction was allowed to proceed for 4.5 h to ensure the complete Ag shell formation. Fabricated Au@Ag nanocuboids were centrifuged at 7500 rpm for 5 min and redispersed in 1 mL of 0.1 M CTAC.

#### Synthesis of nanorattles

0.5 mL as prepared Au@Ag nanocuboids were diluted to 2 mL with water. While magnetically stirred at room temperature, 50 μL of 50 mM freshly prepared AA as a mild reducing agent followed by 10 μL of 1 mM H_2_PdCl_4_ aqueous solution were injected at room temperature. The mixture was left under stirring for 10 min. To increase the extent of Ag shell hollowing, Au@Ag nanocuboids were further titrated with 10 μL of 50 mM AA followed by 10 μL of 1 mM H_2_PdCl_4_ at the time. This process was repeated successively to increase the total amounts of H_2_PdCl_4_ added to the solution. The total volumes of H_2_PdCl_4_ added were 10, 20, 30, 40, 50, 60, 70, 80, 90, 100, 150, 200 μL. The obtained nanorattles were centrifuged at 7500 rpm for 5 min and redispersed in water.

### SERS monitoring of the hydrogenation of 4-nitrothiophenol

The hydrogenation of 4-nitrothiophenol by NaBH_4_ was used as a model reaction to evaluate the catalytic performances of the obtained nanorattles. Nanorattles, obtained with 10, 50, 90, 200 μL of 1 mM H_2_PdCl_4_, were mixed with 1 mL 1 mM 4-NTP aqueous solution and incubated at room temperature overnight to allow 4-NTP molecules adsorption on the surface of nanorattles. Then, 4-NTP functionalized nanorattles were washed with water twice and redispersed in 1 mL of water. The colloid was mixed with 1 mL of 50 mM freshly prepared NaBH_4_ aqueous solution at room temperature. The SERS spectra were recorded under 785 nm laser excitation at a laser power of 1.02 W cm^−2^.

### Characterization and instrumentation

The optical extinction spectra of colloidal nanoparticles were recorded at room temperature on a Shimadzu UV-2600 Plus spectrophotometer, equipped with an integrating sphere in plastic cuvettes of 1 cm optical path length. The structural characterization was made by transmission electron microscopy (TEM) using a JEOL JEM-1400Flash microscope operating at 120 kV. The morphology was investigated by JEOL JSM-IT500HR scanning electron microscope (SEM). Energy dispersive X-ray spectroscopy (EDX) measurements were obtained on SEM equipped with EDAX APEX detector. Powder X-ray diffraction (XRD) patterns of the samples were obtained at room temperature for 1 h with the scattering angle 2*θ* range from 35° to 80°, using a benchtop Miniflex-600 powder X-ray diffractometer (Cu Kα, *λ* = 1.5418 Å). Raman spectra were recorded on a pre-configured QE Pro Raman portable spectrophotometer coupled with 785 nm laser excitation in 1 cm quartz cuvette. Analysis of Pd-content in obtained colloids was performed on SpectroBlue-FMT36 ICP OES spectrometer.

## Conclusions

In conclusion, we have synthesized Au@Ag nanocuboids and used a facile GRR mediated by co-reduction for their transformation into Pd-rich nanorattles to modify their structural, optical, and catalytic properties. It was shown that titration of Au@Ag nanocuboids with various volumes of H_2_PdCl_4_ solution in the presence of AA changed overall morphology of the nanoparticles and their composition. During the early stage dominated by deposition and alloying processes, extinction peak red-shifted over broad spectral range and its intensity increased, reaching the maximum at 850 nm. The enhancement of LSPR peak intensity is a result of Au@Ag/Pd nanorattles dimensions increase and return of Ag atoms, initially constituting the shell, into the frame in the process of co-reduction with Pd by AA. Incorporation of strong plasmonic silver in the outer part of nanorattles prevented plasmon dumping at low concentrations of Pd-precursor in reaction mixture. We consider the successive addition of H_2_PdCl_4_ a key factor in the synthetic protocol of Au@Ag/Pd nanorattles characterized by enhanced LSPRs. Four Pd-rich nanorattle colloids obtained at different stages of GRR were utilized to monitor the reduction reaction of 4-NTP to 4-ATP by SERS. Experimental results demonstrated that as the Pd : Ag atomic ratio increased from 0.018 to 0.286 the reaction time was reduced from 40 min to 14 min. This uniquely enhanced plasmonic and catalytic properties endow these Pd-rich nanorattles with great potential in SERS substrates and nanocatalysts for *in situ* observation and acceleration of many chemical transformations.

## Conflicts of interest

There are no conflicts to declare.

## Supplementary Material

RA-011-D1RA06109G-s001

## References

[cit1] Mukherjee S., Libisch F., Large N., Neumann O., Brown L. V., Cheng J., Lassiter J. B., Carter E. A., Nordlander P., Halas N. J. (2013). Nano Lett..

[cit2] Mukherjee S., Zhou L. A., Goodman A. M., Large N., Ayala-Orozco C., Zhang Y., Nordlander P., Halas N. J. (2014). J. Am. Chem. Soc..

[cit3] Kim Y., Smith J. G., Jain P. K. (2018). Nat. Chem..

[cit4] Christopher P., Xin H. L., Linic S. (2011). Nat. Chem..

[cit5] Boerigter C., Campana R., Morabito M., Linic S. (2016). Nat. Commun..

[cit6] Marimuthu A., Zhang J. W., Linic S. (2013). Science.

[cit7] Jain P. K., Huang X. H., El-Sayed I. H., El-Sayed M. A. (2008). Acc. Chem. Res..

[cit8] Halas N. J., Lal S., Chang W. S., Link S., Nordlander P. (2011). Chem. Rev..

[cit9] Xie W., Herrmann C., Kompe K., Haase M., Schlucker S. (2011). J. Am. Chem. Soc..

[cit10] Quiroz J., Barbosa E. C. M., Araujo T. P., Fiorio J. L., Wang Y. C., Zou Y. C., Mou T., Alves T. V., de Oliveira D. C., Wang B., Haigh S. J., Rossi L. M., Camargo P. H. C. (2018). Nano Lett..

[cit11] Lim B., Jiang M. J., Tao J., Camargo P. H. C., Zhu Y. M., Xia Y. N. (2009). Adv. Funct. Mater..

[cit12] Wang Y., Xie S. F., Liu J. Y., Park J., Huang C. Z., Xia Y. N. (2013). Nano Lett..

[cit13] Huang H. W., Wang Y., Ruditskiy A., Peng H. C., Zhao X., Zhang L., Liu J. Y., Ye Z. Z., Xia Y. N. (2014). ACS Nano.

[cit14] Watt J., Young N., Haigh S., Kirkland A., Tilley R. D. (2009). Adv. Mater..

[cit15] Niu Z. Q., Peng Q., Gong M., Rong H. P., Li Y. D. (2011). Angew. Chem., Int. Ed..

[cit16] Tian N., Zhou Z. Y., Yu N. F., Wang L. Y., Sun S. G. (2010). J. Am. Chem. Soc..

[cit17] Wang F., Li C. H., Sun L. D., Wu H. S., Ming T. A., Wang J. F., Yu J. C., Yan C. H. (2011). J. Am. Chem. Soc..

[cit18] Deng Y. J., Tian N., Zhou Z. Y., Huang R., Liu Z. L., Xiao J., Sun S. G. (2012). Chem. Sci..

[cit19] Collins G., Schmidt M., O'Dwyer C., McGlacken G., Holmes J. D. (2014). ACS Catal..

[cit20] De Marchi S., Nunez-Sanchez S., Bodelon G., Perez-Juste J., Pastoriza-Santos I. (2020). Nanoscale.

[cit21] Jing H., Zhang Q. F., Large N., Yu C. M., Blom D. A., Nordlander P., Wang H. (2014). Nano Lett..

[cit22] Swearer D. F., Zhao H. Q., Zhou L. N., Zhang C., Robatjazi H., Martirez J. M. P., Krauter C. M., Yazdi S., McClain M. J., Ringe E., Carter E. A., Nordlander P., Halas N. J. (2016). Proc. Natl. Acad. Sci. U. S. A..

[cit23] Aslam U., Chavez S., Linic S. (2017). Nat. Nanotechnol..

[cit24] Joplin A., Jebeli S. A. H., Sung E., Diemler N., Straney P. J., Yorulmaz M., Chang W. S., Millstone J. E., Link S. (2017). ACS Nano.

[cit25] Moon Y., Mai H. D., Yoo H. (2017). ChemNanoMat.

[cit26] Robatjazi H., Zhao H. Q., Swearer D. F., Hogan N. J., Zhou L. N., Alabastri A., McClain M. J., Nordlander P., Halas N. J. (2017). Nat. Commun..

[cit27] Jing H., Large N., Zhang Q. F., Wang H. (2014). J. Phys. Chem. C.

[cit28] DeSantis C. J., Weiner R. G., Radmilovic A., Bower M. M., Skrabalak S. E. (2013). J. Phys. Chem. Lett..

[cit29] Habas S. E., Lee H., Radmilovic V., Somorjai G. A., Yang P. (2007). Nat. Mater..

[cit30] Lu C. L., Prasad K. S., Wu H. L., Ho J. A. A., Huang M. H. (2010). J. Am. Chem. Soc..

[cit31] Lee Y. W., Kim M., Kim Z. H., Han S. W. (2009). J. Am. Chem. Soc..

[cit32] Wang F., Sun L. D., Feng W., Chen H. J., Yeung M. H., Wang J. F., Yan C. H. (2010). Small.

[cit33] Li G. G., Wang Z. X., Wang H. (2020). ChemNanoMat.

[cit34] Gonzalez E., Arbiol J., Puntes V. F. (2011). Science.

[cit35] Sun Y. G., Xia Y. N. (2002). Science.

[cit36] Sun Y. G., Xia Y. N. (2004). J. Am. Chem. Soc..

[cit37] Chen J. Y., McLellan J. M., Siekkinen A., Xiong Y. J., Li Z. Y., Xia Y. N. (2006). J. Am. Chem. Soc..

[cit38] Lu X. M., Tuan H. Y., Chen J. Y., Li Z. Y., Korgel B. A., Xia Y. N. (2007). J. Am. Chem. Soc..

[cit39] Xia X. H., Wang Y., Ruditskiy A., Xia Y. N. (2013). Adv. Mater..

[cit40] Cobley C. M., Xia Y. N. (2010). Mater. Sci. Eng., R.

[cit41] Rycenga M., Cobley C. M., Zeng J., Li W. Y., Moran C. H., Zhang Q., Qin D., Xia Y. N. (2011). Chem. Rev..

[cit42] Richard-Daniel J., Boudreau D. (2020). ChemNanoMat.

[cit43] Sutter E., Jungjohann K., Bliznakov S., Courty A., Maisonhaute E., Tenney S., Sutter P. (2014). Nat. Commun..

[cit44] Li J. M., Sun X. J., Qin D. (2016). ChemNanoMat.

[cit45] Li J. M., Liu J. Y., Yang Y., Qin D. (2015). J. Am. Chem. Soc..

[cit46] Zhang H., Liu Z. K., Kang X. L., Guo J., Ma W. L., Cheng S. (2016). Nanoscale.

[cit47] Chen J. Y., Wiley B., McLellan J., Xiong Y. J., Li Z. Y., Xia Y. N. (2005). Nano Lett..

[cit48] Zhu X. Z., Xu J., Zhang H., Cui X. M., Guo Y. Z., Cheng S., Kan C. X., Wang J. F. (2020). Chem. Sci..

[cit49] Xu J., Yun Q. R., Wang C. S., Li M. M., Cheng S., Ruan Q. F., Zhu X. Z., Kan C. X. (2020). Nanoscale.

[cit50] Jing H., Wang H. (2015). Chem. Mater..

[cit51] Jing H., Wang H. (2014). CrystEngComm.

[cit52] Polavarapu L., Zanaga D., Altantzis T., Rodal-Cedeira S., Pastoriza-Santos I., Perez-Juste J., Bals S., Liz-Marzan L. M. (2016). J. Am. Chem. Soc..

[cit53] Russo L., Merkoci F., Patarroyo J., Piella J., Merkoci A., Bastus N. G., Puntes V. (2018). Chem. Mater..

[cit54] Jiang R. B., Chen H. J., Shao L., Li Q., Wang J. F. (2012). Adv. Mater..

[cit55] Gawande M. B., Goswami A., Felpin F. X., Asefa T., Huang X. X., Silva R., Zou X. X., Zboril R., Varma R. S. (2016). Chem. Rev..

[cit56] Liu K. K., Tadepalli S., Tian L. M., Singamaneni S. (2015). Chem. Mater..

[cit57] Genc A., Patarroyo J., Sancho-Parramon J., Bastus N. G., Puntes V., Arbiol J. (2017). Nanophotonics.

[cit58] Mahmoud M. A., El-Sayed M. A. (2010). J. Am. Chem. Soc..

[cit59] Deeb C., Zhou X., Plain J., Wiederrecht G. P., Bachelot R., Russell M., Jain P. K. (2013). J. Phys. Chem. C.

[cit60] Lohse S. E., Murphy C. J. (2012). J. Am. Chem. Soc..

[cit61] Yavuz M. S., Cheng Y. Y., Chen J. Y., Cobley C. M., Zhang Q., Rycenga M., Xie J. W., Kim C., Song K. H., Schwartz A. G., Wang L. H. V., Xia Y. N. (2009). Nat. Mater..

[cit62] Prodan E., Radloff C., Halas N. J., Nordlander P. (2003). Science.

[cit63] Wang Z. L., Ahmad T. S., ElSayed M. A. (1997). Surf. Sci..

[cit64] Sieradzki K. (1993). J. Electrochem. Soc..

[cit65] Huang J. F., Zhu Y. H., Lin M., Wang Q. X., Zhao L., Yang Y., Yao K. X., Han Y. (2013). J. Am. Chem. Soc..

