# Supplementary material for: Palladium-rich plasmonic nanorattles with enhanced LSPRs via successive galvanic replacement mediated by co-reduction
Source: RSC Adv. 2021 Dec 16;11(63):40112–9. doi: 10.1039/d1ra06109g (PMC9044558; doi:10.1039/d1ra06109g)
Supplement: RA-011-D1RA06109G-s001 [file RA-011-D1RA06109G-s001.pdf]

Electronic Supplementary Material (ESI) for RSC Advances.
This journal is © The Royal Society of Chemistry 2021

Supporting Information

Palladium-rich Plasmonic Nanorattles with Enhanced LSPRs *via* Successive Galvanic Replacement Mediated by Co-reduction

Mariia Ivanchenko[a], Andrew J. Evangelista[a], Hao Jing*[a]

[a]Department of Chemistry and Biochemistry, George Mason University, Fairfax, Virginia 22030, USA

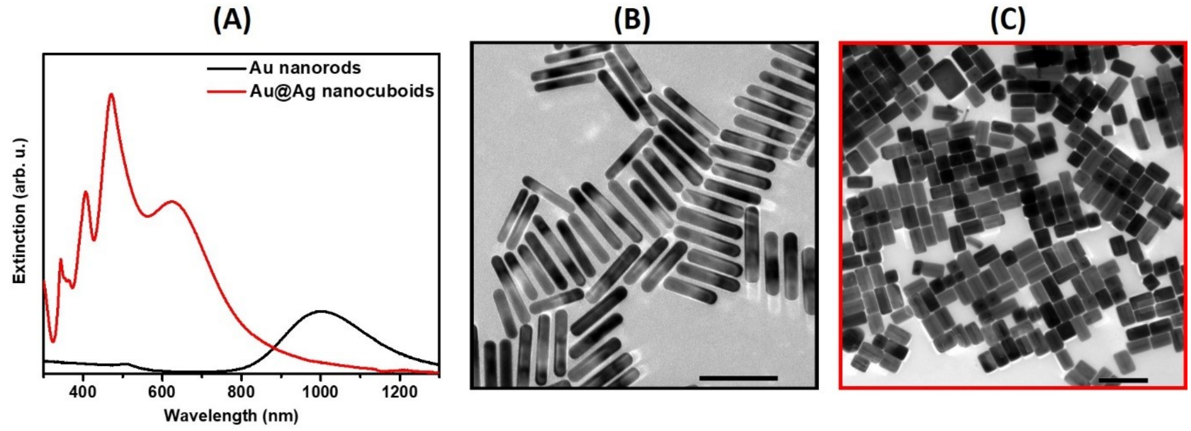

Fig. S1. (A) Extinction spectra of Au NRs and Au@Ag nanocuboids colloidal solutions. (B) TEM image of Au NRs. Scale bar corresponds to 100 nm. (C) TEM image of Au@Ag nanocuboids. Scale bar corresponds to 200 nm.

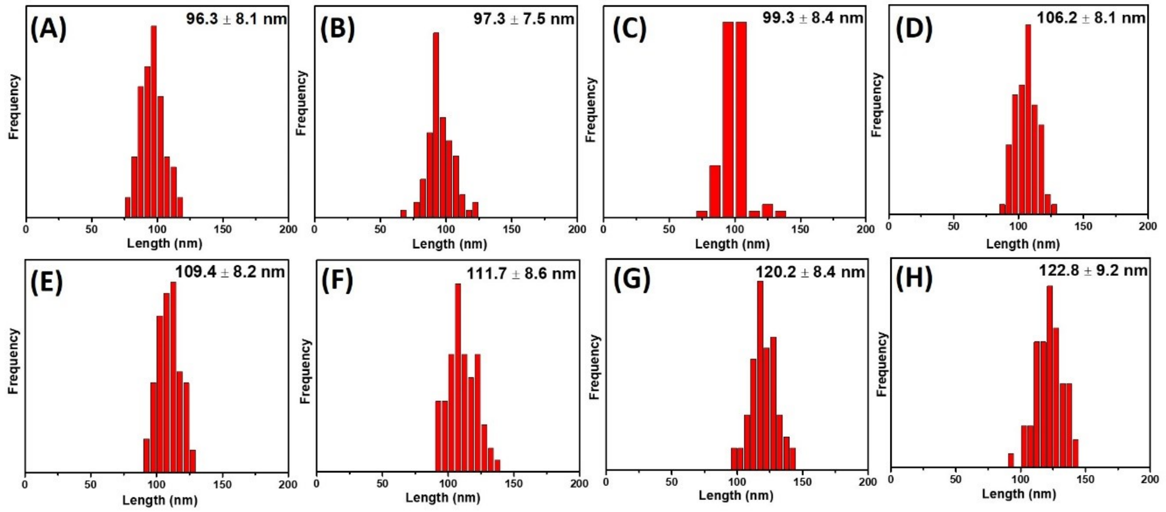

Fig. S2. Length distributions of (A) Au@Ag nanocuboids and (B-H) Au@Ag_Pd nanorattles obtained using (B) 10 µL, (C) 30 µL, (D) 50 µL, (E) 70 µL, (F) 90 µL, (G) 150 µL, and (H) 200 µL of 1 mM H_2PdCl_4 obtained from analysis of TEM images.

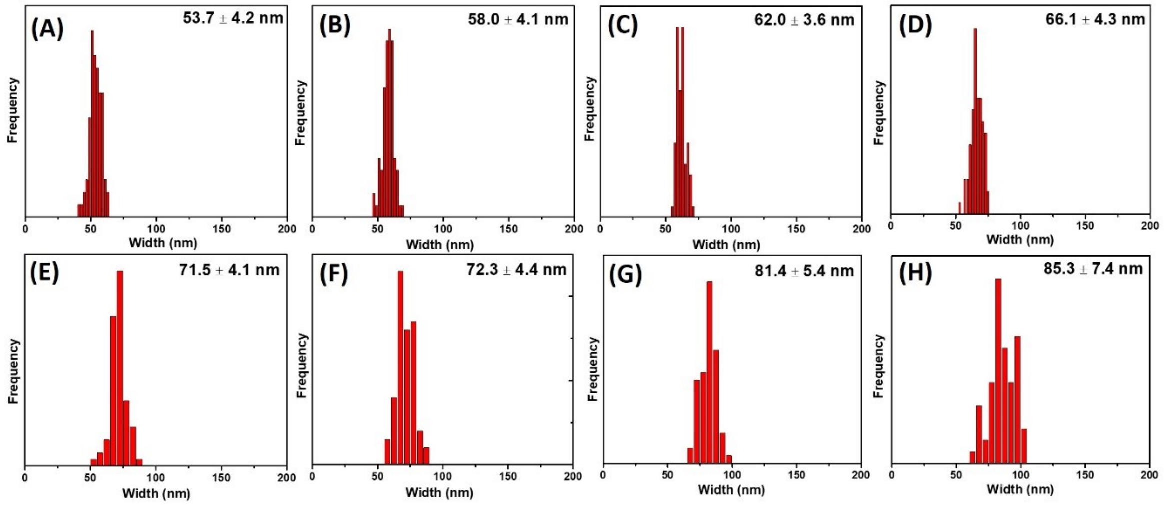

Fig. S3. Width distributions of (A) Au@Ag nanocuboids and (B-H) Au@Ag_Pd nanorattles obtained using (B) 10 µL, (C) 30 µL, (D) 50 µL, (E) 70 µL, (F) 90 µL, (G) 150 µL, and (H) 200 µL of 1 mM H_2PdCl_4 obtained from analysis of TEM images.

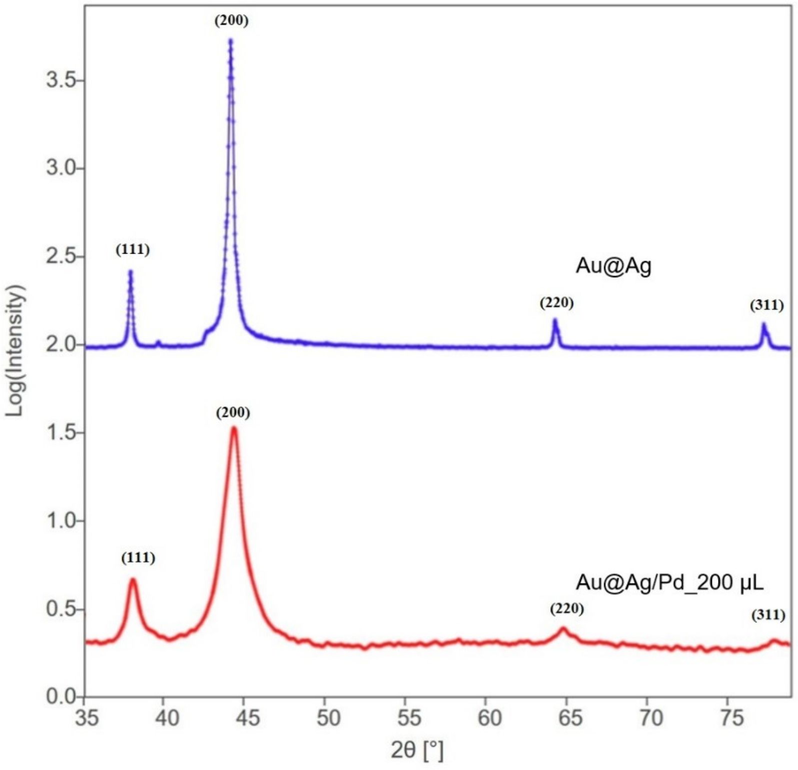

Fig. S4. XRD patterns of Au@Ag nanocuboids and Au@Ag/Pd nanorattles.

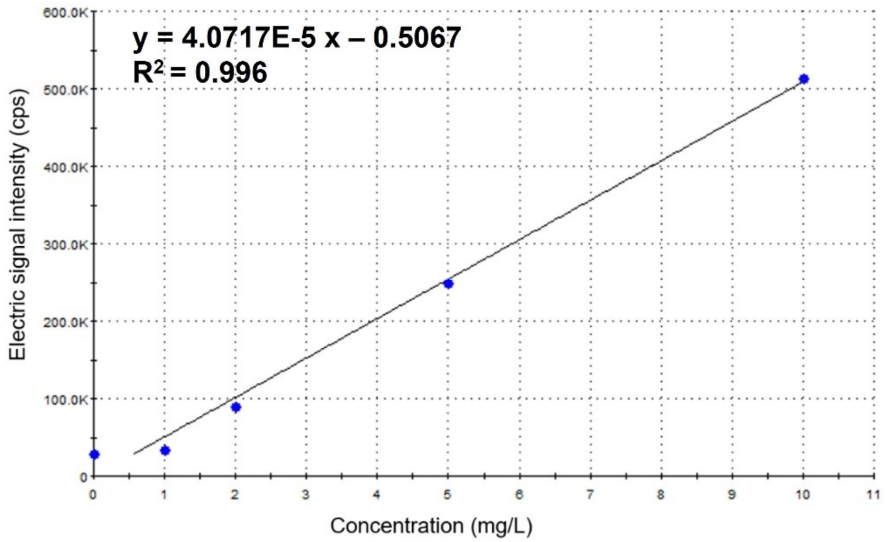

Fig. S5. Five-point calibration curve for ICP-OES measurements of Pd-content.

Table S1. The leaching study of Pd active sites in Au@Ag/Pd nanorattles obtained using 200 µL of 1 mM H_2PdCl_4 during 4-NTP to 4-ATP conversion based on ICP-OES results. (standard deviation is 0.40 mg/L)

**Sample**	**Concentration of Pd in nanorattles**
Before reaction	21.64 mg/L
After reaction	19.51 mg/L

Table S2. Comparison of the catalytic performance based on the kinetic constants (*k*) of the multicomponent plasmonic nanomaterials (including Au, Ag and Pd) of various morphologies for the conversion of the 4-NTP to 4-ATP obtained from SERS monitoring.

Nanostructure	*k*	Pd content	Ref.
Au@Ag/Pd nanorattles	0.044 s^{-1}	mole%(Pd) =1.6%	This work
	0.069 s^{-1}	mole%(Pd) =8.0%	
	0.088 s^{-1}	mole%(Pd) =13.6%	
	0.146 s^{-1}	mole%(Pd) =20.9%	
AuAg:Pd concave nanolayers	5.5 × 10^{-3} s^{-1}	mole%(Pd) = 4.8%	1
	1.0 × 10^{-2} s^{-1}	mole%(Pd) = 9.6%	
Au@AgPd core/shell nanoflowers	6.64×10^{-3} s^{-1}	wt%(Pd) = 45.3%	2

References

1 W. S. Huang, I W. Sun, C. C. Huang. *J. Mater. Chem. A*, 2018, **6**, 13041-13049.

2 Y. Lai, L. Dong, R. Liu, S. Lu, Z. He, W. Shan, F. Geng, Y. Cai, J. Liu. *Chin. Chem. Lett.,* 2020, **31**, 9, 2437-2441.
